# Optical Fluorescence Imaging of Native Proteins Using a Fluorescent Probe with a Cell-Membrane-Permeable Carboxyl Group

**DOI:** 10.3390/ijms23105841

**Published:** 2022-05-23

**Authors:** Jung Min Kim, Young-Mi Kang

**Affiliations:** 1 BK21 FOUR R&E Center for Environmental Science and Ecological Engineering, Korea University, 145 Anam-ro, Seongbuk-gu, Seoul 02842, Korea; 2 Department of Orthopaedic Surgery, Yonsei University College of Medicine, 50-1 Yonsei-ro, Seodaemun-gu, Seoul 03722, Korea; ymkang@yuhs.ac

**Keywords:** carboxyl fluorescent dye, single native proteins of interest, highly efficient fluorescence labeling, cell-permeable fluorescent dye, bioorthogonal reactions, nickel-nitrilotriacetic acid bead assay

## Abstract

Although various methods for selective protein tagging have been established, their ap plications are limited by the low fluorescent tagging efficiency of specific terminal regions of the native proteins of interest (NPIs). In this study, the highly sensitive fluorescence imaging of single NPIs was demonstrated using a eukaryotic translation mechanism involving a free carboxyl group of a cell-permeable fluorescent dye. In living cells, the carboxyl group of cell-permeable fluorescent dyes reacted with the lysine residues of acceptor peptides (AP or AVI-Tag). Genetically encoded recognition demonstrated that the efficiency of fluorescence labeling was nearly 100%. Nickel-nitrilotriacetic acid (Ni-NTA) beads bound efficiently to a single NPI for detection in a cell without purification. Our labeling approach satisfied the necessary conditions for measuring fluorescently labeled NPI using universal carboxyl fluorescent dyes. This approach is expected to be useful for resolving complex biological/ecological issues and robust single-molecule analyses of dynamic processes, in addition to applications in ultra-sensitive NPIs detection using nanotechnology.

## 1. Introduction

Fluorescence-based methods are widely used to investigate interactions of native proteins of interest (NPIs) and intracellular molecules [1]. The modulation of NPI activity in vitro and in vivo as well as the identification of mechanisms of action are important for the development of treatments [2,3]. As precise fluorescent tool for analyses of biological/ecological systems, organic dyes are well-characterized small-molecule fluorescent labeling agents (<1 kDa) [4,5,6,7]. Historically, fluorescent proteins (FPs) have been used as genetically encoded labels [8,9,10]. The maturation efficiency tends to be high in proteins, such as green fluorescent protein (EGFP), which are selectively tagged in the complex environment of living cells such that fluorescence can be observed only after the polypeptide chain is folded [10,11]. Therefore, although the labeling efficiency is close to 100%, the fluorescence intensity depends on the molecular brightness and number of FPs in a limited functional modality [12]. Typical methods for the identification of expressed proteins are limited for the detection of purity, molecular weight, and structure within a picogram concentration range, owing to the purification processes.

Selective tagging of cell surface proteins is one approach to label cellular structures [13,14] and viral components in live cells [15,16]. However, improvements are required for fluorescent tagging [17] and labeling of specific terminal regions of NPIs [18].

Previous studies confirmed that a simple bead-based assay for sensitively quantifying the amount of native state green fluorescent protein using Ni-NTA (nickel-nitrilotriacetic acid)-modified microbead particles [19]. The nickel-nitrilotriacetic acid (Ni-NTA) bead analysis can detect these NPIs on the basis of the interaction between hexahistidine (6× His)-tag residues of considerably low concentration NPIs in their native structure state [20,21]. Moreover, a Ni-NTA bead analysis can be verified that a target protein can be quickly identified in its native state with high sensitivity [19]. Protein labeling is facilitated by a bioconjugation reaction of specific terminal amino acid residues of NPIs that form covalent bonds with various carboxyl group dyes. In particular, biotin ligase (BirA) derived from *Escherichia coli* was used to catalyze the activation of the free carboxyl group on the lysine side chain to an adenylated ester within the 15-amino acid “Acceptor Peptide (AP or AVI-Tag)” peptide sequence commonly found in living cells [22,23]. Escherichia coli biotin ligase (BirA) is utilized in numerous biotechnological applications, including protein labelling [24,25,26].

The natural substrate of BirA is the biotin carboxyl carrier protein (BCCP). Before smaller tags were discovered, a protein needed to be fused to the entire BCCP to be targeted [27]. A protein fused by BCCP can be recognized by biotin molecules in vivo and attach to it [28]. A few other small tags have been used before AVI-Tag, but AVI-Tag is the most efficient so far [29]. We have focused on expanding the biotin analogues specificity of BirA to incorporate fluorescent dyes containing free carboxyl groups (ATTO 565-biotin, 5(6)-carboxyfluorescein [FAM 56], sulfo-Cyanine3 [Cy3-COOH]). However, despite their excellent photophysical properties, organic dyes [30] and nanoparticles [31,32] usually cannot be utilized for site-specific labeling in live cells.

Furthermore, examples of enzyme-mediated site-specific protein labeling in vivo have been reported for endogenous mammalian proteins, such as *O*^6^-alkylguanine-DNA alkyltransferase (AGT) and dihydrofolate reductase (DHFR) [33]. These tags are used for background labeling of endogenous counterparts in mammalian cells and the non-covalent interactions that rely on labeling DHFR and FK506-binding protein (FKBP) in vivo, resulting in rapid dissociation and consequent signal degradation [34].

Over the past few decades, nanotechnology innovations have led to the development of various single-molecule technologies [35,36]. The high-sensitivity detection of fluorescent molecules has provided kinetic and thermodynamic information about the molecular activity to establish structure–function relationships at the single molecule level [37,38]. Single-molecule detection requires [39]: (1) high sensitivity, enabling the detection of weak signals from individual molecules without purification, and (2) a high signal-to-noise ratio and image quality. Additionally, in our study, BirA-based labeling was complemented by single-molecule-level imaging [26], and this approach can be applied to any protein of interests.

Biotinylation and biotin/streptavidin affinity techniques are essential biosensing tools [40,41] in proteomics [40,42]. Proximity-dependent biotin identification (BioID) is a powerful tool to identify novel protein–protein and proximity-based interactions in living cells [43,44]. In this technique, the biotin ligase is fused to an NPI and expressed in vitro in the desired cells, where it biotinylates proximal endogenous proteins. Despite its advantages for biological studies, BioID has limited capacity for selective site-specific labeling in living cells [45,46]. ATTO 565-Biotin, used in this study, enables efficient labeling in living cells by combining site-specific proteomics and efficient immobilization by inter actions between biotin/streptavidin and native proteins [47,48].

Here, we investigated an ultra-sensitive fluorescent labeling protein that exhibits high specificity to NPI within the cell via genetic fusion of a cell-permeable carboxyl group dye with an AVI-Tag sequence. In this study, a high-efficiency (100%) fluorescence labeling was developed for proteins, wherein the binding of a dye/molecule containing a carboxyl group to the terminal site of an NPI in living cells was investigated using Ni-NTA bead assay.

## 2. Results

### 2.1. Free Carboxyl Group-Containing Fluorescent Dye Ligates the Lysine (K) Residue of the AVI-Tag in Living Cells

**Figure 1:**
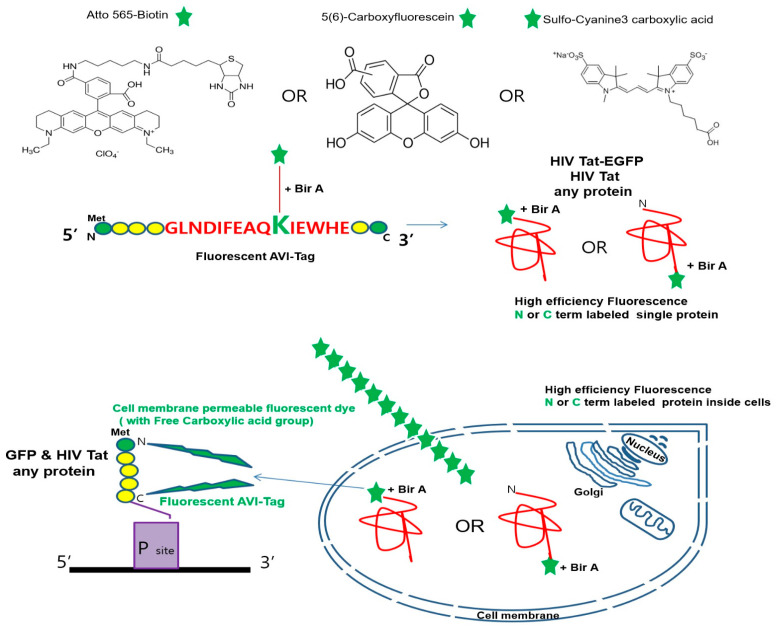
Structure of ATTO 565-biotin, 5(6)-carboxyfluorescein (FAM 56), and sulfo-Cyanine3 carboxylic acid (sulfo-Cy3-COOH) used in this study. Scheme showing selective labeling in cells of single native state proteins using a fluorescent dye containing at least one free carboxyl group (green star) bound to biotin co-expressed biotin ligase (BirA). Using an engineered fluorescent acceptor peptide (AP or AVI-Tag) for the N- or C-terminal, site-specific labeling of proteins with FAM 56 and sulfo-Cy3-COOH were obtained. First, the fluorescently labeled AVI-Tag consisting of 15 amino acids serves as a substrate for bacterial BirA. Second, the AVI-Tag, containing a central lysine, specifically reacts with the fluorescent probes via its amino group (methionine (green circle), amino acid chain (yellow circle) with the assistance of BirA. Finally, the protein of interest can be fluorescently labeled at the N- or C-terminal using the fluorescent AVI-Tag that serves as a substrate for BirA in living cells.

In addition to the technological advances related to fluorescent-labeled proteins, this study provides a conceptual design for tagging using dyes attached to the C-terminus of the protein. On the basis of the intracellular labeling requirements for the co-translational integration of the carboxyl group of a fluorescent molecule in relation to our strategy, the structure of a cell-membrane-permeable free carboxyl fluorescent dye used for the expression of a fluorescent-labeled NPI (e.g., the HIV-1 Tat protein) is provided in Figure 1.

First, a carboxyl group was attached to a free lysine residue of AP (or AVI-Tag; GLN DIFEAQKIEWHE) comprising 15 amino acids. The NPI was fluorescently labeled by the reaction between the free carboxyl group of the dye and an amine-reactive group in the peptide. Next, amide binding by a cell-membrane-permeable free carboxyl fluorescent dye was achieved by cell permeabilization for the integrated fluorescence of NPIs in mammalian cells. In this study, we focused on the integration of various fluorescent dyes (ATTO 565-biotin red (FLD Ex: 565 nm, Em: 590 nm), FAM 56; green (FLD Ex: 492 nm, Em: 517 nm), and sulfo-Cy3-COOH; red (FLD Ex: 548 nm, Em: 563 nm)) into NPIs that can be directly derived by efficient fluorescent tagging through selective reactions in cells. *E. coli* BirA could serve as a catalyst for the N- or C-terminal labeling of an NPI bound to a free carboxylic acid handle within the AVI-Tag sequence (Figure S1).

To identify the effects of a high signal-to-noise ratio on the binding of various structural analogs of specific free carboxyl groups to the AP or AVI-Tag, the effects of R-phycoerythrin (PE)-labeled streptavidin alone (Figure S2) and BirA alone (Figure S3) were evaluated via size exclusion chromatography–high-performance liquid chromatography (SEC-HPLC). In the analysis of PE-labeled streptavidin, peaks at 6.547 min (fluorescence detection (FLD) with excitation–emission (Ex/Em) wavelengths of 488/575 nm (blue line)) and 5.576 min (UV at 280 nm (black line)) correspond to the fluorescent tag and target protein (280 nm). The two peaks for BirA alone did not correspond to the fluorescent tag or target protein.

### 2.2. Analysis of the Free Carboxylic Acid Dye Uptake Subcellular Localization and Its Fluorescent Labeling

**Figure 2:**
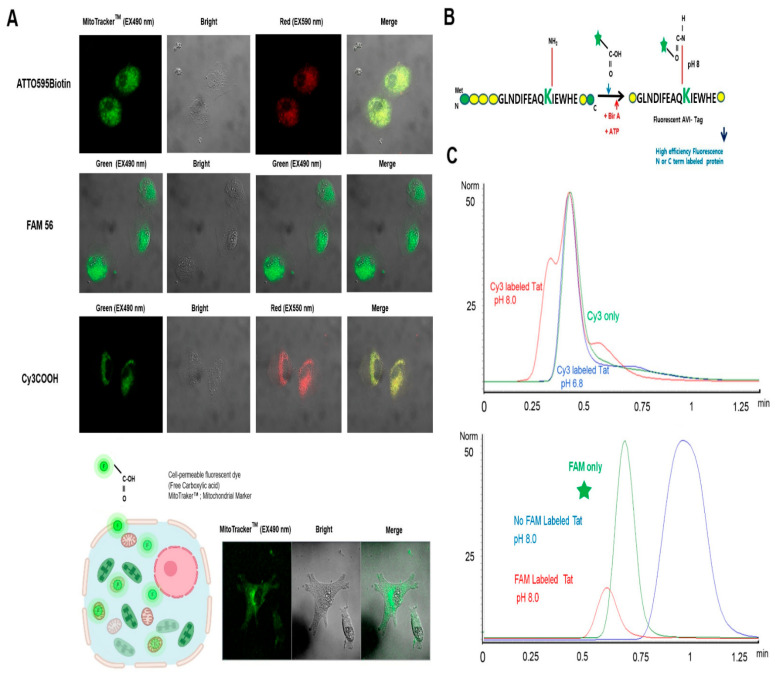
Intracellular distributions of ATTO 565-biotin, 5(6)-carboxyfluorescein (FAM 56), and sulfo-cyanine3 carboxylic acid (sulfo-Cy3-COOH) in the Saos-2 osteosarcoma cell line (Saos-2) observed by fluorescence imaging, bright-field microscopy, dark-field microscopy, and fluorescence imaging of samples stained with MitoTracker™. (**A**) The excitation wavelengths for ATTO 565-biotin, FAM 56, and sulfo-Cy3-COOH were 565, 492, and 550 nm, respectively. The free carboxyl groups of fluorescent dyes used for single-fluorescence imaging analysis (MitoTracker™ (green), FLD Ex: 490 nm, Em: 516 nm). Scale bar = 20 μm. (**B**) HPLC traces (the highest peak corresponding to the retention time of 7.21 min as see in Figure 4) showing BirA- and ATP-dependent ligation of free carboxyl fluorescent dyes to a synthetic acceptor peptide (GLNDIFEAQKIEWH; the acceptor lysine (Lys, K) is green). (**C**) Analysis of various pH conditions for fluorescently labeled Tat via UPLC. The distinct peak of Cy3-labeled Tat at pH 8.5 (red) at 0.25 min reflects its apparent separation that was obtained at pH 5.8 (blue) and using Cy3 carboxylic dye only (green); FLD, Ex: 548 nm/Em: 563 nm. Overlay of UPLC profiles obtained by FAM 56-labeled Tat at pH 8.0 (red) at 0.5 min reflects its apparent separation that was obtained at pH 8.5 (blue) and using FAM 56 dye only (green); FLD, Ex: 492 nm/Em: 517 nm.

First of all, we investigated the capability of the cell-permeable free carboxyl fluorescent molecules to penetrate into osteosarcoma cells. To examine their mitochondria-targeting ability, osteosarcoma cells were incubated with MitoTracker as a control group and ATOO565Biotin, 5(6) carboxyfluorescein, and sulfo-cyanine 3 carboxylic acid (Figure 2A) as the experiment group, followed by confocal imaging. MitoTracker is a commercially available fluorescent dye (Invitrogen/Molecular Probes) that, like the aforementioned dyes, labels mitochondria within live cells utilizing the mitochondrial membrane potential. However, MitoTracker is chemically reactive, linking to thiol groups in the mitochondria. The dye becomes permanently bound to the mitochondria and thus remains after the cell dies or is fixed. Thus, it can be used in these experiments in which multiple labeling localizes cellular targeting.

Representative images of fluorescent dyes whose free carboxyl fluorescent dyes penetrated the cytosol are shown in Figure 2A. The three aforementioned fluorescent dyes freely penetrated the cell membrane, and their permeabilization into the cytosol was detected by the green fluorescence signal ((green), FLD Ex: 480 nm, Em: 540 nm, red signal (red), FLD Ex: 540 nm, Em: 590 nm) and DIC (optical; bright light) images, as shown in Figure 2A. Figure 2B provides a schematic illustration of a fluorescent-labeled HIV Tat protein containing a free carboxyl group. Its strategy shows how the free carboxyl group of the fluorescent dye allows the NPI to be selectively AVI-Tagged in living cells by genetic encoding in vivo/in vitro.

Next, we detected the Tat labeling efficiency of sulfo-Cy3-COOH, which was analyzed and determined using UPLC resolution under optimized pH 8.0-dependent Cy3-labeling conditions (Figure 2C). The high-intensity peak observed for the negative control group containing sulfo-Cy3-COOH dye alone suggests that Tat was not separated via UPLC, unlike the high-intensity peak for the group labeled with the sulfo-Cy3-COOH dye in 5 min, where Tat could be detected.

As shown in Figure 2C, *E. coli* BirA was utilized to label the free carboxyl group of the fluorescent derivative. The conjugation reaction of FAM 56 at the N- or C-terminus was unambiguously identified using BirA-dependent high-level fluorescein (FAM 56) labeling, for which the peak obtained via UPLC (1 min, blue line) was well separated.

As shown in Figure 2C, the peak separation results for fluorescein in the negative control group distinctly indicate a peak representing the free carboxyl group fluorescent derivative alone (0.5 min, green line) and another UPLC-separated peak for BirA-independent fluorescein (FAM 56) labeling (5 min, red line), suggesting efficient expression of the target-specific label of the carboxyl group-containing dye in the experimental group.

Furthermore, the biotin-binding peak was distinctly identified by in vitro labeling with PE-streptavidin alone, which followed the same procedure as that for the ATTO 565-biotin dye, after treating the designated biotin-binding PE-streptavidin alone in the control group (Figure S2). On the basis of the strategy described in Figure S4, we identified the peak for the BirA protein. The biotin/streptavidin binding reaction was used to deter mine the specificity of AVI-Tag residue labeling. The HPLC-separated peak (7.5 min, blue line) for the BirA-dependent labeling of the FAM 56 dye and biotin and the HPLC-separated peak (4.5 min, red line) for PE-streptavidin labeling differed from the peaks for sulfo-Cy3-COOH (Figure S4).

### 2.3. Detection of the Fluorescently Labeled NPI

**Figure 3:**
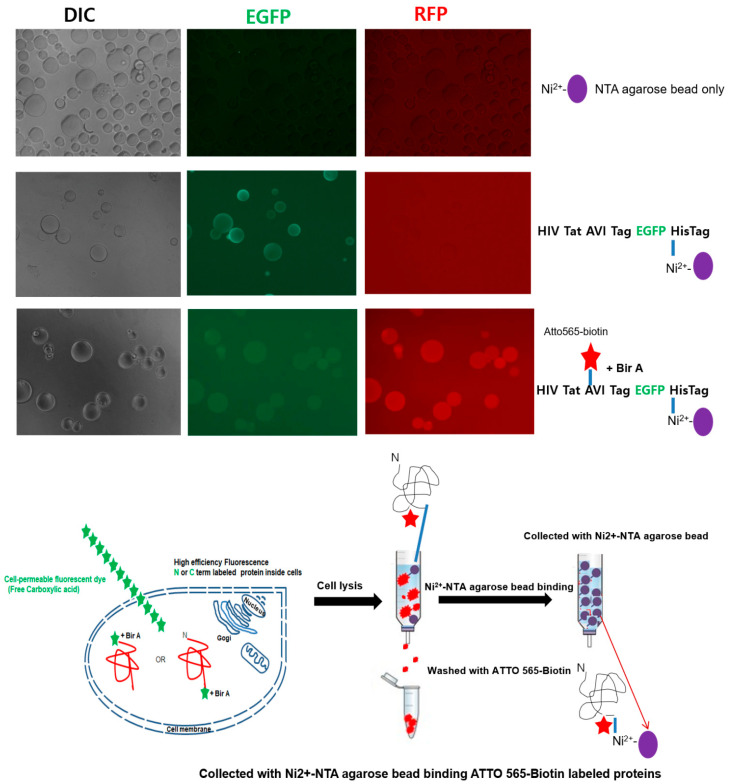
Binding between Ni-NTA beads and the free carboxyl group of the fluorescent (ATTO 565-biotin, red star or cell-permeable fluorescent dye with carboxylic acid, green star)-labeled Tat-EGFP protein, as observed under a fluorescence microscope. Binding between single NTA (nickel-nitrilotriacetic acid) agarose beads (nanoparticles, purple circle) and ATTO 565-biotin-labeled Tat-EGFP was detected. Fluorescence images of beads were obtained after incubating with ATTO 565-biotin-labeled Tat-EGFP, followed by detection through the free carboxyl acid handle using biotin ligase (BirA). Fluorescence images of the green fluorescence signal (green laser; FLD, Ex: 480 nm, Em: 540 nm), red signal (red laser; Ex: 540 nm/Em: 590 nm), and DIC (optical; bright light) were obtained using a fluorescence microscope; comparisons are only made among beads under the same fluorescence exposure time with a single NTA. The purified Tat-EGFP protein concentration was confirmed by analyzing the fluorescence signal from the combination of conditions with the same fluorescence exposure time to a single NTA agarose bead (purple circle). A single Tat-EGFP-binding microbead can be detected with any instrument or filter set compatible with Tat-EGFP detection: FLD, Ex: 480 nm/Em: 540 nm.

To verify the high sensitivity of ATTO 565 biotin labeling, we captured the FP in its native state through the interaction between Ni-NTA and the 6× His residue of a target protein using Ni-NTA-modified microbead single particles that did not require purification to remove the cell lysate. The ATTO 565-biotin-labeled Tat-binding fluorescence images of these beads were analyzed via fluorescence microscopy (Figure 3). Bead-based detection of fluorescently labeled NPI was applied to the target protein in its native state at very low concentrations [19]. Fluorescence images of beads were obtained using BirA to detect ATTO 565-biotin-labeled Tat-(EGFP) bound to the free carboxylic acid handle within the AVI-Tag sequence. The in vitro Ni-NTA agarose assay involved a simple quantitation method using agarose bead particles (diameter: 45–165 µm) modified with NPI-sensitive Ni-NTA for quantitative detection. This NPI-sensitive quantitative detection method was comparable to Ni-NTA-modified agarose beads that immediately bound to the NPI through a reaction between Ni-NTA and 6× His-tag fused to EGFP (compared with the corresponding control; 6× His-tagged EGFP) as a control group.

This strategy ensured that the protein was not denatured upon being attached to the surface of the bead particle [19]. Additionally, the intensity of the fluorescence signal from the 6× His tag fused to EGFP on the bead was closely related to the amount of ATTO 565-biotin-labeled Tat in its NPI status. In Figure 3, green-filter fluorescence signals (green; FLD Ex: 480 nm, Em: 540 nm) are shown for Ni-NTA beads alone and the ATTO 565-biotin-unlabeled Tat-EGFP control group, in addition to the red-filter fluorescence signals (red; FLD Ex: 540 nm, Em: 590 nm) for ATTO 565-biotin-labeled Tat-EGFP. Notably, the interaction between the intracellular lysate and the 6× His residue of the target protein on the surface of the Ni-NTA bead facilitated the capture and detection of the FP in its native state with high sensitivity. Our selective 6× His-tag method was capable of labeling NPIs utilizing a known enzyme dependence in mammalian cells while retaining cell surface protein activity and specificity. Our selective 6× His-tag method is capable of labeling NPIs utilizing a Bir A ligase dependence in mammalian cells while retaining cellular protein activity and specificity. Likewise, in vitro enzyme-based, site-specific protein labeling and BioID strategies are examples of selective protein labeling through genetic targeting [43,45]. In this method, labeling by highly specific gene targeting can be spatially controlled. As proposed previously [19], our bead assay can overcome the limited efficiency of broad-targeted NPI labeling without compromising function and can address the limitations of conventional denatured protein detection methods and quantitative image analysis.

### 2.4. Identification of Fluorescence-Labeled NPIs Separated by SEC-HPLC According to LC–MS Spectra

**Figure 4:**
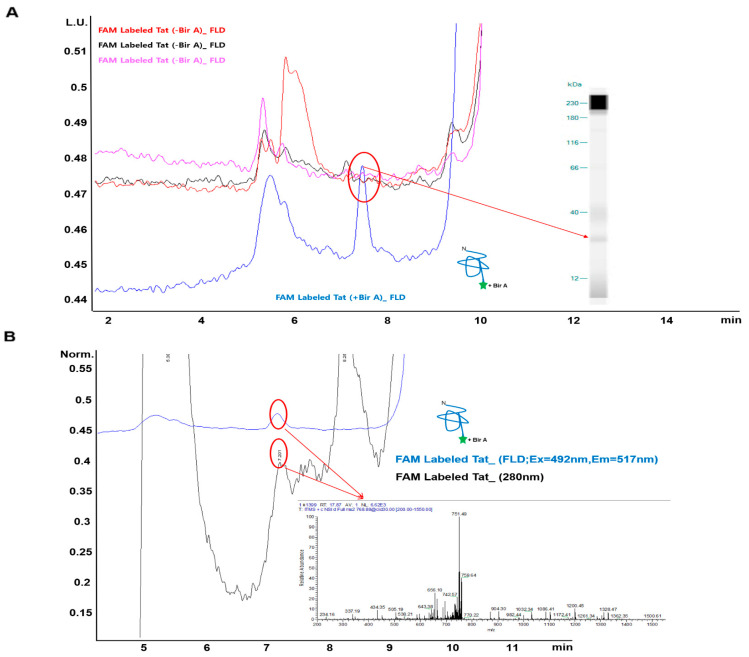
UPLC and LC–MS analysis of 5(6)-carboxyfluorescein (FAM 56)-labeled Tat protein identification. HeLa cells treated with free carboxyl fluorescent dyes were analyzed for free carboxyl fluorescent (Sulfo-Cyanine3 (Csulfo-Cy3), FAM 56) Tat by HPLC, and fluorescent Tat proteins were identified by LC–MS. (**A**) HPLC traces (highest peak corresponding to the retention time of 7.21 min) showing BirA- and ATP-dependent ligation of free carboxyl fluorescent dyes to a synthetic acceptor peptide. The 7.21 min peak showed clear fluorescence detection (FLD, Ex: 492 nm/Em: 517 nm, blue line) and UV detection (280 nm, black line), and the separated peak at 7.21 min showed a band consistent with the FAM 56-labeled Tat peptide as determined by Western blotting. (**B**) LC–MS spectrum showing the mass of purified AP-free carboxyl-acid-conjugated Tat fluorescent (FAM) Tat protein containing a C-terminal AVI-Tag that was recovered from HeLa extracts using Ni-affinity spin columns. Identification of Tat proteins using LC–MS data relies on the peptide map from a Tat sequence matched to a target sequence database. The following peptide sequences in the Tat protein matched with the spectrum measurement (AHQNSQTHQASLSK). Each data point represents the average of three experiments.

As shown in Figure 4, LC–MS was performed to identify and analyze the target molecule, Tat, labeled with the cell-permeable carboxyl group-conjugated dye. The labeling efficiency of the carboxyl group dye for the NPI was evaluated through a comparative analysis of the FAM 56 dye alone, BirA-independent experimental groups, and FAM 56-labeled Tat (Figure 2C).

The UPLC peak area for FAM 56-labeled Tat was larger than those for other groups, suggesting that direct use of FAM 56 within the cell improves the labeling efficiency. SEC-HPLC revealed a unique peak for FAM 56-labeled Tat at 7.201 min (indicative of mediated labeling) (Figure 4). The separated and purified carboxyl group fluorescent-labeled Tat protein was subjected to MS/MS, followed by nanoelectrospray ionization (nESI) on an LTQ Orbitrap Velos (Thermo Scientific, Inc., San Jose, CA, USA) coupled inline to HPLC.

This peak at 7.201 min was successfully separated and purified using LC–MS and positively confirmed to be the Tat protein by Western blotting (Figure 4A and Figure S5). The AHQNSQTHQASLSK sequence consistent with the intact Tat peptide was detected in the Orbitrap at a resolution of 60,000 (Figure 4B and Figure S6). These results verified the target specificity of carboxyl dyes detected by LC–MS, utilizing the peptide map obtained from a matching database that targets the highly purified, 6× His-tagged FAM 56-labeled Tat sequence, tracked using chromatography.

### 2.5. The Universal Carboxy Group Fluorescent Dye Mediated the Expression of Fluorescence-Labeled NPIs at the Single-Molecule Level

**Figure 5:**
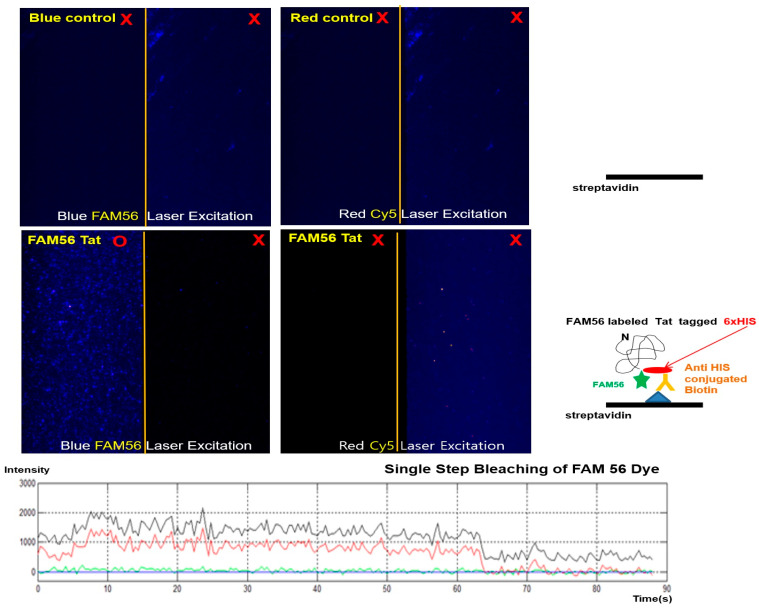
Detection of fluorescent Tat expression using the universal carboxylic group fluorescent dye at the single-molecule level. Non-specific binding tests with or without streptavidin on the surface were performed in T50 buffer. We used 50 buffer (pH 8.0) for all non-specific binding tests, unless otherwise specified. Compared with spots expressing (red O and red X) the streptavidin-specific biotinylated fluorescent Tat protein on the surface, relatively few spots indicating non-specific binding were observed in the T50 buffer (positive control, upper). Detection of the 5(6)-carboxyl fluorescein (FAM 56) counterpart (sample, lower) immobilized on quartz slides coated with streptavidin was performed. The FAM 56-labeled Tat (sample, lower, FLD, Ex: 492 nm/Em: 517 nm) was immobilized on quartz slides. Blue laser (FAM 56 spots; blue circles) is shown as a TIRF image of immobilized FAM 56-labeled Tat proteins on the surface. Red laser (Cy5 spots; no circles, FLD, Ex: 620 nm/Em: 670 nm) is shown as a TIRF image of immobilized FAM 56-labeled Tat proteins on the surface. Scale bar = 5 μm. All experiments were performed at room temperature (22 ± 1 °C). Traces of single FAM 56 dye bleaching (red and black line, twice times) observed more than once for the FAM 56 labeled Tat proteins in the time-fluorescent intensity TIRF image.

To further evaluate the single fluorescently labeled NPI, the double-tagged single Tat protein was extracted from HeLa cells to confirm single fluorescent Tat expression (Figure 5).

Currently, single-molecule detection is crucially involved in a wide range of applications. In previous studies [49], this approach has been efficiently utilized for the structural analysis of target proteins at the single-molecule level, along with a dual-label control containing a C-terminal AVI-Tag for N-terminal fluorescent probe activity.

To confirm the translation efficiency of the FAM 56-labeled protein, we prepared a single-fluorescent FAM 56 dye with an optimized N-terminal recognition motif and a C-terminal interaction partner fused to an AVI-Tag. Single-molecule imaging was per formed using biotin double-labeled Tat on polyethylene-glycol-coated quartz slides. Single-molecule fluorescence images were acquired continuously using a total internal reflection fluorescence (TIRF) microscope [49]. The single molecule was immobilized on a quartz slide for all non-specific binding experiments unless otherwise designated. Figure 5 shows a representative fluorescent single-molecule image processed with the IDL script to identify 5-FAM spots and to extract the 5-FAM intensity of individual spots (red circles). The single dye bleaching event was also observed for the FAM 56-Tat protein in TIRF image (Figure 5), confirming that each fluorescent spot corresponded to a labeled Tat protein.

We determined the conditions required to measure fluorescently labeled synthesized proteins using a universal carboxy group fluorescent dye at the single-molecule level, which are useful for both in vitro and in vivo studies. We demonstrated a highly efficient strategy for fluorescently labeling NPIs without compromising their function by introducing various carboxyl dyes/biotin for NPI detection in its native states.

## 3. Discussion

We demonstrated that co-translation is possible in living cells using a fluorescent dye with a free carboxyl group to enhance the fluorescent labeling efficiency of NPIs.

Analysis of the fluorescence labeling efficiency of NPIs was performed in a previous study using a simple bead assay to sensitively quantify the amount of green fluorescent protein in its native state using microbead particles modified with Ni-NTA. This assay successfully demonstrated high-efficiency labeling of NPIs without functional sacrifice.

In our study, BirA-based free carboxyl dye labeling successfully established a fluorescent label for low-concentration NPIs with high-sensitivity molecular detection and high labeling efficiency. Fluorescent imaging also confirmed the potential of fluorescent NPIs at the single-molecule level.

In previous reports, BirA-dependent label-based NPIs were combined with AVI-Tag specific sequences for NPI identification, limiting the method to in vitro purified proteins and intracellular cell surface proteins [13,45,50]. In addition, Bir A-based biotinylation techniques using known biotin analogue techniques are not suitable for NPI detection or single-molecule imaging. In a previous study, a labeling reaction for sulfonated Cy5 can be used in the labeling reaction to label proteins dye-to-protein (D/P) ratio over a broad concentration range of over 100-fold was reported [17,51]. In addition, it is known to use a high concentration of organic dye for the D/P ratio in a conjugation reaction using a purified protein [51,52]. High-purity fluorescently labeled natural proteins in eukaryotes are conventional in vitro targets that require complex purification processes. The efficiency of protein conjugation to organic dyes is very low, resulting in low fluorescence. Site-specific labeling of live cells with organic dyes and nanoparticle-bound molecules is not possible. However, in our study, NPI labeling with free carboxylic acid group dyes was based on enzyme binding (e.g., BirA ligase) [53], which can be obtained with higher efficiency than conventional protein conjugation (e.g.,−NH_2_ (amine) group of a lysine or the free −SH (sulfhydryl) group of cysteine) [54,55].

Regarding the localization of distinct patterns, the ratio of fluorescence of the back ground region to the intracellular region is important because it can interfere with specificity for NPIs, thereby reducing fluorescence efficiency. Therefore, we used a locally labeled Mitotracker as a control permanently bound to mitochondria to label mitochondria in living cells using commercially available fluorescent dyes (Invitrogen/Molecular Probes). Mitochondrial markers were identified using cellular NPI of an intracellular penetrating fluorescent dye with a genetically encoded AVI-Tag region to identify matching locations. Our results demonstrate that high signal-to-noise ratios can be obtained after conjugation of free carboxyl groups, demonstrating high fluorescence labeling efficiency and independent targeting of terminal molecules of fluorescent dyes. Therefore, we used a fluorescent dye with a free carboxyl group to improve the fluorescent labeling efficiency of NPI for co-translation in living cells. However, controlling transmission of a fluorescent dye into the cell membrane with a free carboxyl group alone remains challenging. Our method has unlimited potential for versatility because it utilizes a universal carboxyl group that allows for the introduction of optical nanoparticle materials other than fluorescent dyes. It provides new insights into the potential to facilitate biological/ecological analyzes of living organisms.

## 4. Materials and Methods

### 4.1. Materials

FAM 56 dye and ATTO 565-biotin dye were purchased from Sigma-Aldrich Co. (St. Louis, MO, USA). Vivaspin 500 (pore size 0.2 µm) was purchased from Sartorius, Inc. (Göttingen, Germany). Sulfo-Cy3-COOH was purchased from Lumiprobe Corporation (Hallandale Beach, FL, USA). MitoTracker™ Green FM ((green), FLD Ex: 490 nm, Em: 516 nm) and Ni-NTA-agarose beads (pore size 50–150 µm) were obtained from Thermo Fisher Scientific (San Jose, CA, USA). PE-streptavidin, as the positive control for ATTO 565-biotin tagging, was purchased from BD Biosciences (San Jose, CA, USA). Human osteosarcoma (Saos-2) and HeLa cells, obtained from the American Tissue Type Collection (ATCC, Manassas, VA, USA), were grown in Eagle’s minimal essential medium (MEM) (Gibco, Grand Island, NY, USA).

### 4.2. Fluorescence Imaging of the Free Carboxyl Fluorescent Dyes in Living Cells

For expression of FAM 56, ATTO 565-biotin, and sulfo-Cy3-COOH, the medium was replaced with MEM without phenol red and 1% penicillin/streptomycin (WelGENE, Inc., Daegu, Korea) in a 5% CO_2_ atmosphere at 37 °C. Saos-2 cells were grown to 60–90% confluence and then washed once with growth medium (MEM without phenol red), twice with 1× PBS at pH 7.4, and once more with 1× PBS (pH 7.4) alone. The washed cells were incubated for about 3–6 h and then treated with free carboxyl fluorescent dyes (1 M) FAM 56, ATTO 565-biotin, and sulfo-Cy3-COOH (10 μL per 6 × 10^6^ cells in 12 mL of 6 × 10^6^ cells; 1% penicillin/streptomycin-free media) by incubation at 37 °C for 24 h.

### 4.3. Construction of Expression Plasmids

EGFP and the HIV-1 transcription activator protein Tat containing AVI-Tag and 6× His-tag were used as model proteins for evaluation of the free carboxyl fluorescent labeling method. The EGFP expression plasmid was constructed by conventional PCR sub-cloning of the EGFP gene of pEGFP-N1 (Clontech, Mountain View, CA, USA) into pcDNA3.1(+) (Invitrogen, Carlsbad, CA, USA). The HIV Tat plasmid was constructed as follows: the HIV-1 Tat gene was selected from the HIV-1 complete genome (GenBank ID: NC_001802), chemically synthesized (Genscript, Leiden, The Netherlands), and used as a template for PCR amplification. The forward primer was 5′-GGA TCC ATG GAG CCA GTA GAT CCT AGA CTA GAG-3′, and the reverse primer was 5′-GAA TTC TTA TTC GTG CCA TTC GAT TTT CTG AGC CTC GAA GAT GTC GTT CAG ACC CGC GG-3′. BamHI and EcoRI restriction sites were generated using standard restriction cloning methods, and the fragment was inserted into pcDNA3.1+ (Invitrogen). The open reading frame of the *E. coli* BirA gene was amplified from *E. coli* genomic DNA using PCR and introduced into the pcDNA3 vector [56].

### 4.4. Labeling of Living Cells for Co-Translational Tagging of Target Proteins and Free Carboxyl Fluorescent Dyes

For co-translational fluorescent labeling of target proteins, co-transfection of the target plasmid and BirA plasmid was performed. The cell medium was replaced with MEM without phenol red and 1% penicillin/streptomycin (WelGENE, Inc., Daegu, Republic of Korea) in a 5% CO_2_ atmosphere at 37 °C. HeLa cells were grown to 60–90% confluence and then washed once with growth medium (MEM without phenol red), twice with 1× PBS at pH 7.4, and once with 1× PBS (pH 7.4) alone. The washed cells were incubated in free carboxyl fluorescent dyes (Sigma-Aldrich, St. Louis, MO, USA; 10 μL per 6 × 10^6^ cells in 12 mL of 6 × 10^6^ cells; using penicillin/streptomycin-free media) for about 3–6 h, and then transfected with each target protein expression plasmid and the BirA plasmid (500 ng per 2 × 10^6^ cells) using Lipofectamine 2000 (5 µL; Invitrogen) and incubated at 37 °C for 24 h.

### 4.5. Single Agarose Bead Assay for ATTO 565-Biotin-Labeled Tat-EGFP Protein Detection with Fluorescence Microscopy

For C-terminal ATTO 565-biotin-labeled protein expression, HeLa cells were plated on 6 cm dishes (2 × 10^4^ per dish; Nunc, Denmark) and lysed with freeze–thaw lysis buffer (600 mM KCl, 20 mM Tris-Cl; pH 7.8, and 20% glycerol) according to the Tansey Lab’s ultimate freeze-thaw lysis for mammalian cell protocol [57]. The lysate was centrifuged (15,000 rpm at 4 °C for 15 min), and the clarified supernatant was loaded with binding buffer (200 µL, 50 mM PBS, pH 7.4) and a constant diluted volume (10 μL) of suspended Ni-NTA-agarose beads (pore size 50–150 µm; Thermo Scientific Waltham, MA, USA). After shaking during incubation at 4 °C for 10–30 min under constant bead population, the Ni-NTA agarose bead solutions were with the binding buffer without washing. The amount of 6× His-tagged ATTO 565-biotin-labeled Tat-EGFP precipitated with the 6× His-tagged ATTO 565-biotin-labeled Tat-EGFP-bound beads was assessed by fluorescence microscopy [19].

### 4.6. Liquid Chromatography Analysis of Fluorescent Dyes Labeling of Target Proteins

The HeLa lysate was centrifuged (15,000 rpm at 4 °C for 15 min), and the clarified supernatant was loaded onto HisPur™ Ni-NTA Spin Columns (Pierce Biotechnology, Rockford, IL, USA), followed by the addition of the binding and elution buffers (50 mM sodium phosphate and 300 mM sodium chloride (PBS) without 10 mM imidazole at pH 7.2 and 5.8, respectively). The purified protein was then analyzed by UPLC and SEC on an HPLC system (Agilent Technologies, Inc., Santa Clara, CA, USA) using a Yarra SEC-2000 column (300 × 7.8 mm; Phenomenex, Torrance, CA, USA). For chromatographic separation, solvent A (150 mM NaCl and 50 mM sodium in 50 mM sodium phosphate; pH 6.5) was equilibrated with a linear gradient of solvent B (water with 0.1% TFA, 99.5% purity; Sigma-Aldrich, St. Louis, MO, USA) over 70 min at a flow rate of 1 mL min^−1^. The purity (>95.0%) of the N-terminally labeled protein was confirmed by analytical SEC-HPLC (Agilent Technologies, Inc., Santa Clara, CA, USA). Western blotting was per formed using a Simple Western™ system (WES, ProteinSimple, San Jose, CA, USA) and an anti-penta-His antibody (1:1000 in TBST; Qiagen, Hilden, Germany) for detection (data not shown).

### 4.7. LC–MS Analysis of Labeled Protein

The C-terminal ATTO 565-biotin-labeled Tat was separated by SDS-PAGE, identified by staining with Coomassie Blue, and in-gel digested. For multistage MS/MS experiments, the LC–MS analysis was performed using an LTQ Orbitrap Velos mass spectrometer (Thermo Finnigan, San Jose, CA, USA) equipped with an nESI source to allow simultaneous recording of full-scan mass and collision-induced dissociation (CID) spectra. Data were acquired in data-dependent mode. For peptide mapping of the Tat protein, the CID spectra were compared to the sequence of HIV-1 Tat using Sequest (Bioworks; Thermo Electron Corp., Waltham, MA, USA). PE (control group) is an accessory photosynthetic pigment found in red algae. It exists in vitro as a 240 kDa protein with 23 phycoerythrobilin chromophores per molecule (554061 BD Biosciences).

### 4.8. Detection of Fluorescence and Biotin-Fused Tat

A universal carboxyl fluorescent dye was introduced into living cells to visualize single Tat protein translation. For the freeze–thaw lysis of mammalian cells, Tansey Lab’s ultimate freeze–thaw lysis for mammalian cell protocol [57] was followed, as described above. After protein purification (as described above in “Liquid Chromatography Analysis of Fluorescent Dyes Labeling of Target Proteins”), single-molecule fluorescence images were obtained using a prism and electron-multiplying charge-coupled device (EM-CCD) camera (iXon DV887ECS-BV; Andor Technology, South Windsor, CT, USA) by TIRF microscopy [49]. The light sources were the blue laser (FAM 56; Excelsior-473-5c, Spectra-Physics, Santa Clara, CA, USA) and the red laser (Cy5; Excelsior-635-5c, Spectra-Physics, Tokyo, Japan), and data were analyzed using Visual C++ (Microsoft Corporation, Seattle, WA, USA). FAM 56-labeled Tat protein experiments were performed at 37 °C in imaging buffer [58] (20 mM Tris-HCl, pH 8.0), with Tris-HCl (10 mM, pH 7.5), NaCl (50 mM), and an oxygen scavenger system (1 mM Trolox, 1 mg mL^−1^ glucose oxidase, 0.04 mg mL^−1^ catalase, and 0.4% *w*/*v* glucose) (Sigma-Aldrich, St. Louis, MO, USA). Single-molecule data were analyzed using MATLAB and IDL with EM-CCD exposure times of 300 or 1000 ms to obtain single-molecule time traces or dwell times. The purified 6× His-tagged Tat pro tein was incubated in HeLa cells in a buffer containing bicine (50 mM, pH 8.3), ATP (10 mM), Mg(OAc)_2_ (10 mM), d-biotin, and BirA ligase (0.5 U) (AVI-Tag, Avidity Biosciences, La Jolla, CA, USA) overnight at 4 °C for biotinylation.

### 4.9. Statistical Analysis

All values are expressed as means. Error bars indicate the standard deviation. Graph Adobe CS7 software was used for graphing and statistical analysis.

## 5. Conclusions

We successfully developed a highly efficient strategy to fluorescently label an NPI without compromising its function, using an approach involving various carboxyl dyes/carboxyl biotin. As nickel-nitrilotriacetic acid (Ni-NTA) beads were efficiently bound to a single NPI for detection in living cells, our labeling approach demonstrated high-efficiency fluorescent NPIs using a free carboxyl fluorescent dye. These permeable carboxyl group fluorescent dyes introduced into living ecological systems demonstrate the functionality of ultra-sensitive single-molecule fluorescent technology and may be useful for NPI nanotechnology applications and precision molecular diagnostics.

## 6. Patents

A patent connecting to the protection of this manuscript was registered (KR 10-1957979). Baik Lin Seong and Jung Min Kim are listed as inventors.

## Data Availability

All the data in this paper are expressed as means, and the error bars indicate the standard deviations. For the visualization of the results and statistical analysis, we used the Graph Adobe CS7 software. UniProtKB—A6MI22 (A6MI22_9HIV1_*Human immunodeficiency vi rus 1*).

## Supplementary Materials

The following supporting information can be downloaded at https://www.mdpi.com/article/10.3390/ijms23105841/s1.
