# Supplementary material for: Optical Fluorescence Imaging of Native Proteins Using a Fluorescent Probe with a Cell-Membrane-Permeable Carboxyl Group
Source: Int J Mol Sci. 2022 May 23;23(10):5841. doi: 10.3390/ijms23105841 (PMC9143923; doi:10.3390/ijms23105841)
Supplement: Supplementary file 1 [file ijms-23-05841-s001.zip › ijms-1701254-supplementary/ijms-1701254-supplementary.pdf]

Titles of Supplementary Information

Article

Optical Fluorescence Imaging of Native Proteins Using a Fluorescent Probe With a Cell Membrane-Permeable Carboxyl Group

Jung Min Kim [1*], Young-Mi Kang[2]

[1*] BK21 FOUR R&E Center for Environmental Science and Ecological Engineering, Korea University, Anam-ro145, Seongbuk-gu, Seoul 02842, Republic of Korea

[2] Departmrnt of Orthopaedic surgery, Yonsei University college of medicine, 50-1 Yonsei-ro Seodaemun-gu Seoul 03722, Republic of Korea

[*]Correspondence: Tel; +82-2-3290-4778, erine7.kim@gmail.com,

Supplementary Figure S1: The construction of N-terminus-tagged HIV-1 Tat protein.
Supplementary Figure S2: Analysis of R-phycoerythrin (PE)-labeled streptavidin by SEC-HPLC with PE labeled streptavidin as the control group.
Supplementary Figure S3: Analysis of biotin ligase (BirA) by SEC-HPLC. The analytical SEC-HPLC profiles of BirA as the control group.
Supplementary Figure S4: Comparative analysis of BirA enzyme-dependent labeling of FAM 56 with a carboxyl group structure and biotin.
Supplementary Figure S5: LC-MS spectrum of the purified fluorescein (FAM 56)-labeled Tat.
Supplementary Figure S6: Mass spectrum profile of predigested fluorescein (FAM 56)-labeled Tat protein.

Supplementary Figure Legends

Figure S1. The construction of N-terminus-tagged HIV-1 Tat protein.
Schematic description of the HIV Tat protein labeled with free carboxyl group fluorescent dyes at the N-terminus. The hexahistidine (6×His)-tag was introduced in the C-terminus for Ni-affinity chromatography and carrying Avi-tag at the C-terminus for biotin labeling.

Figure S2. Analysis of R-phycoerythrin (PE)-labeled streptavidin by SEC-HPLC with PE-labeled streptavidin as the control group. Compared to the peaks of PE-labeled streptavidin alone, UV (280 nm, black line) detection at 5.576 min, PE-labeled streptavidin is shown with the highest peak at 5.547 min (8 L.U.) (FLD, Ex: 488 nm/Em: 575 nm, blue line). PE emissions are given in relative light units (LU).

Figure S3. Analysis of biotin ligase (BirA) by SEC-HPLC. The analytical SEC-HPLC profiles of BirA as the control group. SEC-HPLC of N-terminally fluorescence-labeled Tat peaks at 10.565 min (UV 280 nm, blue line, black line) and 11.388 min (FLD, Ex: 492 nm/Em: 517 nm, red line), respectively**.**

Figure S4. Comparative analysis of BirA enzyme-dependent labeling of FAM 56 with a carboxyl group structure and biotin. Detection of the N-terminally biotin-labeled Tat protein as the control was performed using R-phycoerythrin (PE)-labeled streptavidin alone. Positive control PE-labeled streptavidin with BirA spectrum (FLD, Ex: 488 nm/Em: 532 nm, blue line) from FAM 56-labeled Tat without BirA as negative control (FLD, Ex: 492 nm/Em: 517 nm, red line).

Figure S5. LC-MS spectrum of the purified fluorescein (FAM 56)-labeled Tat. HPLC-MS of purified FAM 56-labeled Tat alone (The highest peak corresponding to the retention time (RT) of 17.87 min; 751.49 *m/z* detected) was further analyzed by comparison with the HPLC-MS chemical matching database.

Figure S6. Mass spectrum profile of predigested fluorescein (FAM 56)-labeled Tat protein. The purified FAM 56-labeled Tat protein (The highest peak corresponding to the retention time of 7.21 min in Figure 4) from HeLa cells was further analyzed by LC-MS and western blot. Identification of Tat protein using LC-MS data relies on the peptide map from a Tat sequence target matching database. The following peptide sequences in the Tat protein match with the spectrum measurement. (a) RAHQNSQTHQASLSK, (b) AHQNSQTHQASLSK, and (c) LEPWKHPGSQPK, respectively.

Supporting Data

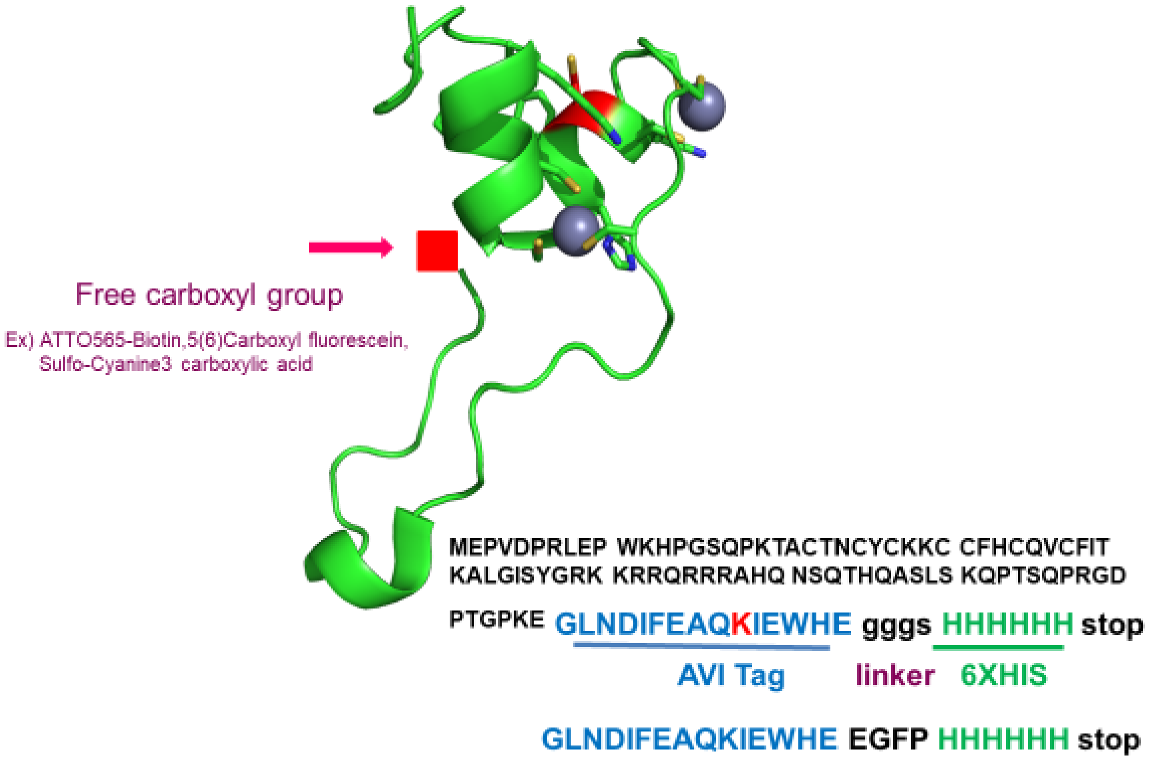

Figure S1. The construction of N-terminus-tagged HIV-1 Tat protein. Schematic description of the HIV Tat protein labeled with free carboxyl group fluorescent dyes at the N-terminus. The hexahistidine (6×His)-tag was introduced in the C-terminus for Ni-affinity chromatography and carrying Avi-tag at the 8-terminus for biotin labeling.

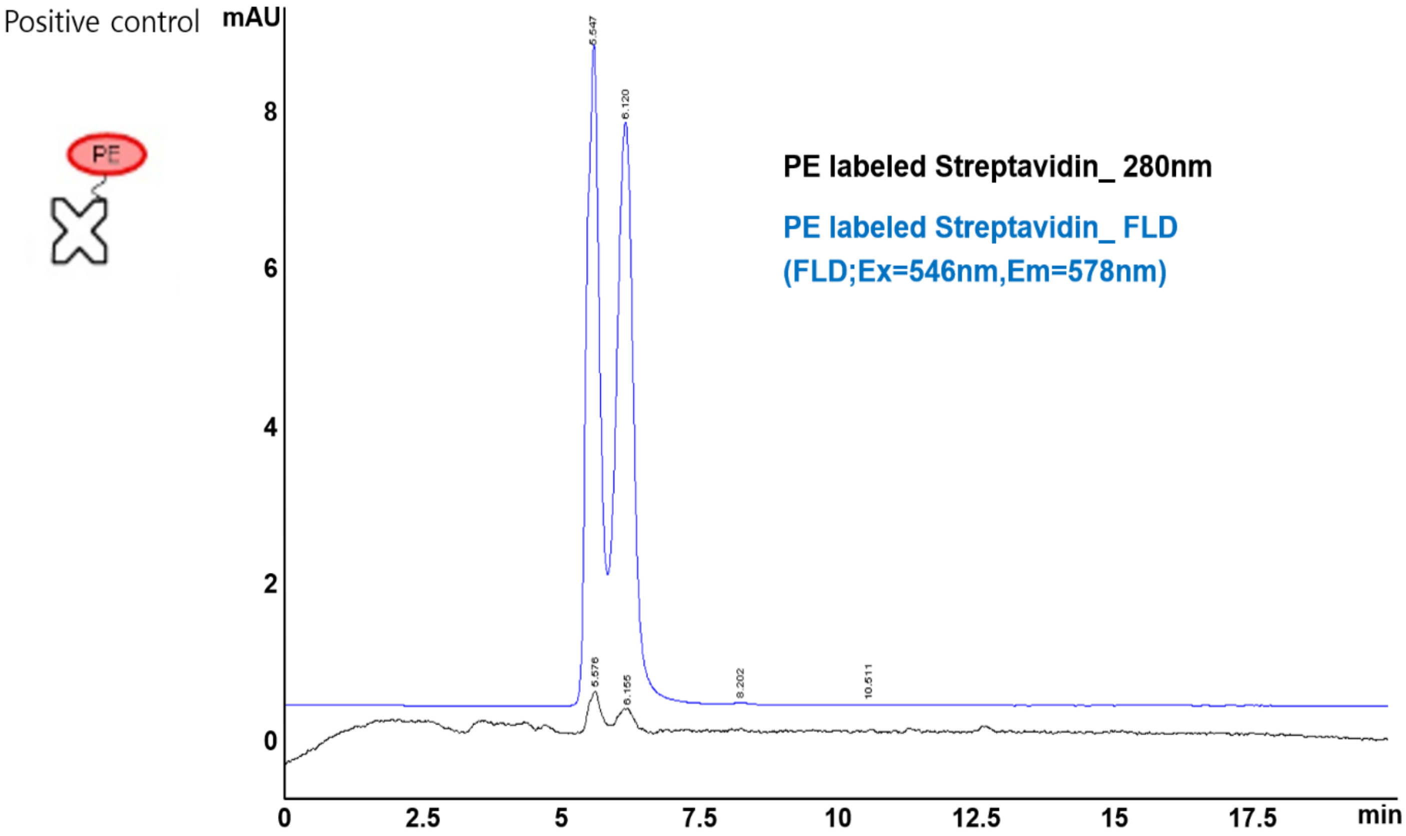

Figure S2. Analysis of R-phycoerythrin (PE)-labeled streptavidin by SEC-HPLC with PE-labeled streptavidin as the control group. Compared to the peaks of PE-labeled streptavidin alone, UV (280 nm, black line) detection at 5.576 min, 1PE-labeled streptavidin is shown with the highest peak at 5.547 min (8 L.U.) (FLD, Ex: 488 nm/Em: 575 nm, blue line). PE emissions are given in relative light units (LU).

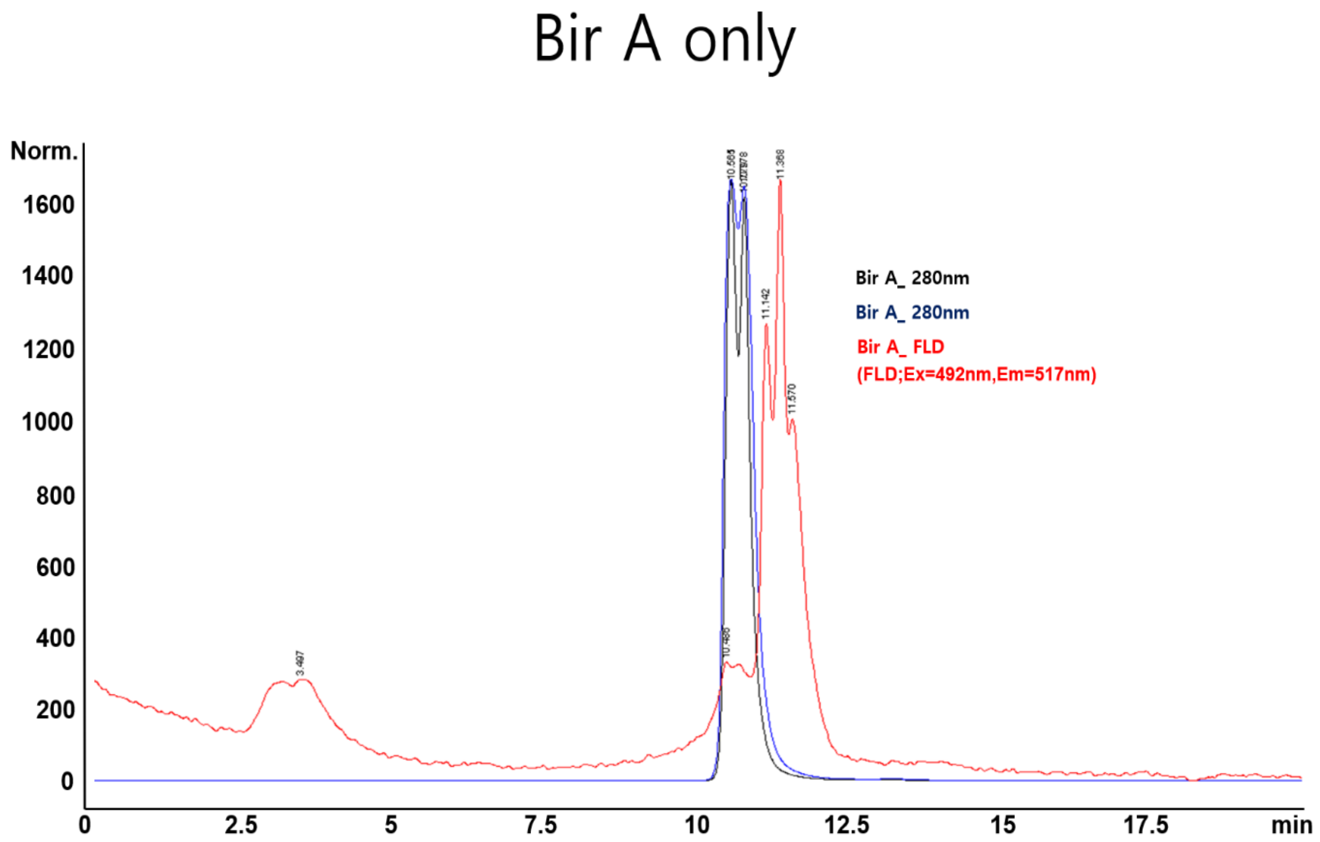

Figure S3. Analysis of biotin ligase (BirA) by SEC-HPLC. The analytical SEC-HPLC profiles of BirA as the control group. SEC-HPLC of N-terminally fluorescence-labeled Tat peaks at 10.565 min (UV 280 nm, blue line, black line) and 11.388 min (FLD, Ex: 492 nm/Em: 517 nm, red line), respectively.

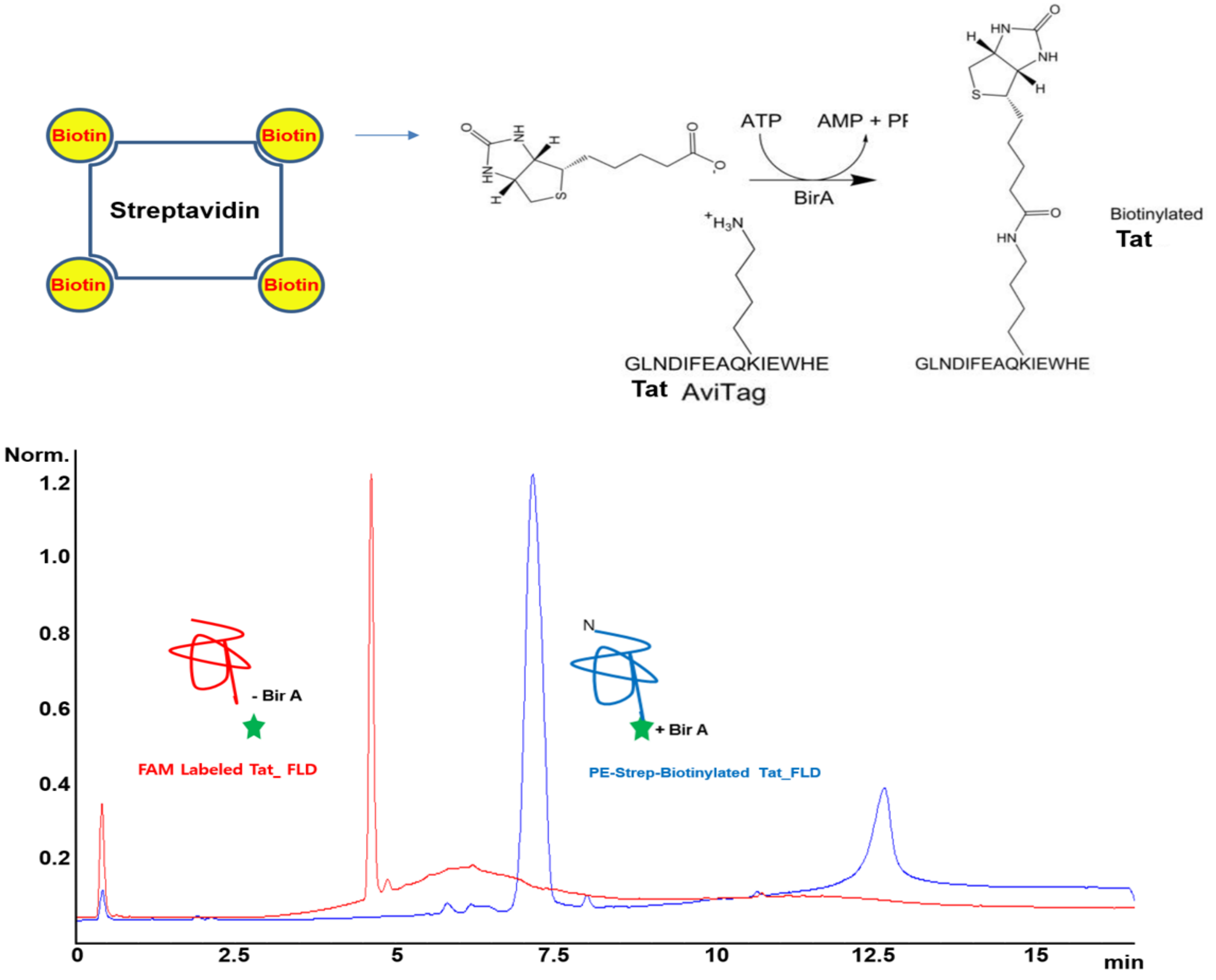

Figure S4. Comparative analysis of BirA enzyme-dependent labeling of FAM 56 with a carboxyl group structure and biotin. Detection of the N-terminally biotin-labeled Tat protein as the control was performed using R-phycoerythrin (PE)-labeled streptavidin alone. Positive control PE-labeled streptavidin with BirA spectrum (FLD, Ex: 488 nm/Em: 532 nm, blue line) from FAM 56-labeled Tat without BirA as negative control (FLD, Ex: 492 nm/Em: 517 nm, red line).

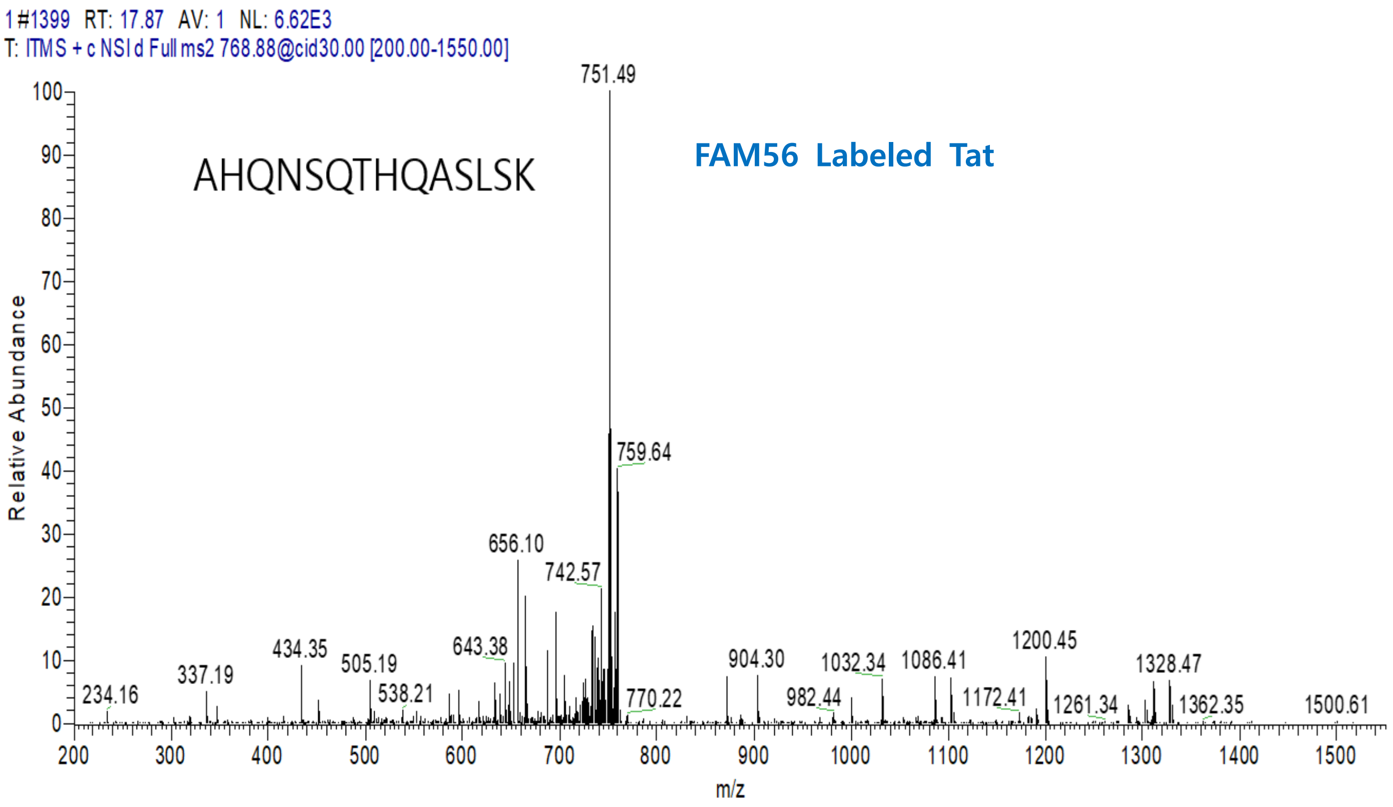

Figure S5. LC-MS spectrum of the purified fluorescein (FAM 56)-labeled Tat. HPLC-MS of purified FAM 56-labeled Tat alone (The highest peak corresponding to the retention time (RT) of 17.87 min; 751.49 *m/z* detected) was further analyzed by comparison with the HPLC-MS chemical matching database.

Sequence of HIV Tat protein.

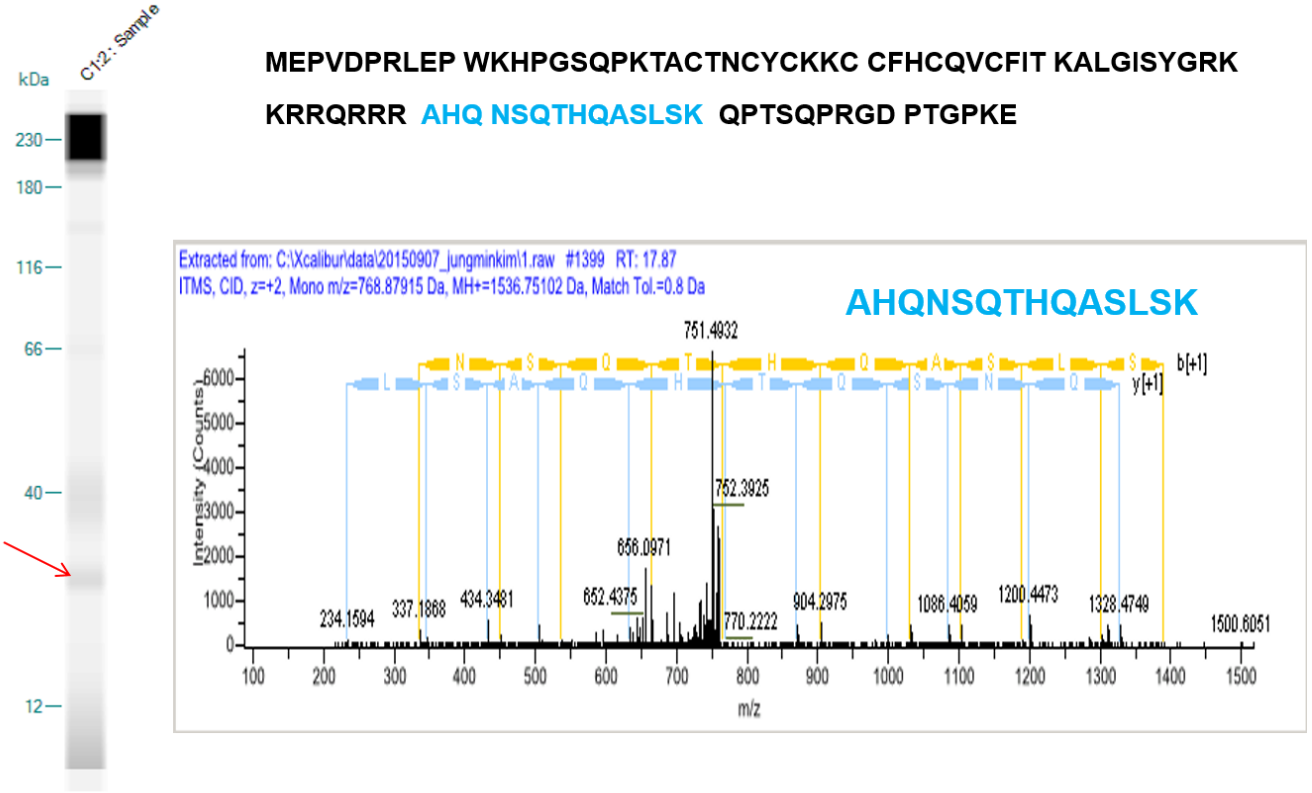

Figure S6. Mass spectrum profile of predigested fluorescein (FAM 56)-labeled Tat protein. The purified FAM 56-labeled Tat protein (The highest peak corresponding to the retention time of 7.21 min in Figure 4) from HeLa cells was further analyzed by LC-MS and western blot. Identification of Tat protein using LC-MS data relies on the peptide map from a Tat sequence target matching database. The following peptide sequences in the Tat protein match with the spectrum measurement. (a) RAHQNSQTHQASLSK, (b) AHQNSQTHQASLSK, and (c) LEPWKHPGSQPK, respectively.
